# Anti-Asian Sentiments During the COVID-19 Pandemic Across 20 Countries: Analysis of a 12-Billion-Word News Media Database

**DOI:** 10.2196/28305

**Published:** 2021-12-08

**Authors:** Reuben Ng

**Affiliations:** 1 Lee Kuan Yew School of Public Policy National University of Singapore Singapore Singapore; 2 Lloyd’s Register Foundation Institute for the Public Understanding of Risk National University of Singapore Singapore Singapore

**Keywords:** racism, COVID-19, anti-Asian sentiments, psychomics, quantitative social science, culture, text as data, xenophobia, digital humanities

## Abstract

**Background:**

US president Joe Biden signed an executive action directing federal agencies to combat hate crimes and racism against Asians, which have percolated during the COVID-19 pandemic. This is one of the first known empirical studies to dynamically test whether global societal sentiments toward Asians have become more negative during the COVID-19 pandemic.

**Objective:**

This study aimed to investigate whether global societal sentiments toward Asians across 20 countries have become more negative, month by month, from before the pandemic (October 2019) to May 2020, along with the pandemic (incidence and mortality rates) and cultural (Hofstede’s cultural dimensions) predictors of this trend.

**Methods:**

We leveraged a 12-billion-word web-based media database, with over 30 million newspaper and magazine articles taken from over 7000 sites across 20 countries, and identified 6 synonyms of “Asian” that are related to the coronavirus. We compiled their most frequently used descriptors (collocates) from October 2019 to May 2020 across 20 countries, culminating in 85,827 collocates that were rated by 2 independent researchers to provide a Cumulative Asian Sentiment Score (CASS) per month. This allowed us to track significant shifts in societal sentiments toward Asians from a baseline period (October to December 2019) to the onset of the pandemic (January to May 2020). We tested the competing predictors of this trend: pandemic variables of incidence and mortality rates measured monthly for all 20 countries taken from the Oxford COVID-19 Government Response Tracker, and Hofstede’s Cultural Dimensions of Individualism, Power Distance, Uncertainty Avoidance, and Masculinity for the 20 countries.

**Results:**

Before the pandemic in December 2019, Jamaica and New Zealand evidenced the most negative societal sentiments toward Asians; when news about the coronavirus was released in January 2020, the United States and Nigeria evidenced the most negative sentiments toward Asians among 20 countries. Globally, sentiments of Asians became more negative—a significant linear decline during the COVID-19 pandemic. CASS trended neutral before the pandemic during the baseline period of October to November 2019 and then plummeted in February 2020. CASS were, ironically, not predicted by COVID-19’s incidence and mortality rates, but rather by Hofstede’s cultural dimensions: individualism, power distance, and uncertainty avoidance—as shown by mixed models (N=28,494). Specifically, higher power distance, individualism, and uncertainty avoidance were associated with negative societal sentiments toward Asians.

**Conclusions:**

Racism, in the form of Anti-Asian sentiments, are deep-seated, and predicated on structural undercurrents of culture. The COVID-19 pandemic may have indirectly and inadvertently exacerbated societal tendencies for racism. Our study lays the important groundwork to design interventions and policy communications to ameliorate Anti-Asian racism, which are culturally nuanced and contextually appropriate.

## Introduction

The COVID-19 crisis has brought with it a disconcerting increase in racism against the Asian community. In the early phase of the pandemic (March 2020), a Singaporean international student in the United Kingdom was physically assaulted in central London [1]. This was an unprovoked attack that the perpetrators justified by saying “we do not want your coronavirus in our country.” Similarly, a Vietnamese curator in London was dropped from an art exhibition, having been told that her presence alone would increase anxiety to those attending the event. She was told that her Asian ethnicity meant that she would be perceived as a carrier of the coronavirus [2].

In the web space, there has been a rise in vitriol and hate speech toward Asians: L1ght found a 900% growth in hate speech towards Asians on Twitter [3]. In the political realm, barbed appellations—“Chinese Virus” and “kung flu” [4]—from government leaders have equated a *whole* ethnicity with the deadly virus and disparaged them, consequently drawing out a fear of Asians. Moreover, the Pew Research Centre found that 58% of Asian Americans reported that it has become more common for people to express racist or racially insensitive views about Asians than before the coronavirus outbreak [5]. Three in 10 Asian American adults have been subject to slurs or jokes because of their race or ethnicity since the outbreak began. In view of the alarming increase in anti-Asian racism, US president Joe Biden signed an executive order on January 26, 2021, directing federal agencies to combat xenophobia toward the Asian American community in the hope of reversing the 2-fold issue of increasing unemployment and hate crimes [6].

Apart from several cross-sectional studies and numerous commentaries [7-10], there are few empirical studies that have assessed the development of anti-Asian sentiments across the pandemic. Our study is one of the first to track anti-Asian sentiments, month by month, from before the pandemic (October 2019) to during the pandemic (May 2020) across 20 countries. We investigated whether global societal sentiments toward Asians across 20 countries have become more negative month by month from before the pandemic (October 2019) to May 2020, as well as the pandemic (incidence and mortality rates) and cultural (Hofstede’s cultural dimensions) predictors of this trend.

We selected Hofstede’s dimensions [11] as they are one of the most widely used measures of cultural values, and are relevant to this study for 3 reasons. First, they provide multiple dimensions from which a culture can be understood. Second, they are widely recognized and understood, demonstrating our study’s comparability and contribution to prior literature. Third, they are based on a national-level understanding of culture, which has been shown to differ from individual-level culture [12], and therefore compatible with the scope of our study as we observe racial sentiments at the national level across 20 countries.

Hofstede’s cultural dimensions [13] are as follows: *individualism* is “the degree to which individuals are supposed to look after themselves or remain integrated into groups, usually around the family”; *masculinity* is “the distribution of emotional roles between the genders (…); it opposes ‘tough’ masculine to ‘tender’ feminine societies”; *uncertainty avoidance* is “the extent to which a culture programs its members to feel either uncomfortable or comfortable in unstructured situations”; *power distance* is “the extent to which the less powerful members of organizations and institutions accept and expect that power is distributed unequally.”

Our study is significant in 3 ways. Conceptually, we contribute to race relations research by analyzing how an extraordinary event, such as the COVID-19 pandemic, may dynamically influence societal sentiments toward an ethnic group. Second, we extend the field of cultural studies by examining the impact of culture on anti-Asian sentiments across 20 countries during a pandemic. Practically, understanding the cultural underpinnings lays the groundwork for designing policy interventions to reduce it, as studies show the malleability of cultural frames [14]—our study provides the cultural considerations to do this effectively.

We hypothesize that societal sentiments toward Asians have become more negative as the pandemic unfolded from October 2019 to May 2020 (hypothesis 1). Second, we tested the factors associated with the hypothesized rise in anti-Asian sentiments during the pandemic. Most opinion-editorials posited that the prevalence of new cases and incidence of new deaths have been associated with increasingly negative racial sentiments (pandemic hypothesis). However, we argue that racism is more deep-seated and is influenced by culture, history, and exclusionary policies such as the Chinese Exclusion Act of 1882 [15]. Discernibly, sociological scholarship reported that cultural factors such as masculine ideals and individualistic traits are associated with racism [16]. Against this background, we hypothesize that both pandemic variables (COVID-19 prevalence and mortality rates) and cultural variables—measured with Hofstede’s cultural dimensions—are associated with negative ethnic sentiments during the pandemic (Hypothesis 2).

## Methods

### Study Design

#### Data Set

We used the news on the web corpus [17], the largest cross-cultural database with 12 billion words ingested from over 30 million articles—found in over 7000 web-based newspapers and magazines—across 20 countries in six continents: North America (United States and Canada), Asia (Bangladesh, Hong Kong, India, Malaysia, Pakistan, Philippines, Singapore, Sri Lanka), Africa (Ghana, Kenya, Nigeria, South Africa, and Tanzania), the Caribbean islands (Jamaica), Europe (Ireland and the United Kingdom), and Oceania (Australia and New Zealand). This data set was created with funding from the National Science Foundation (NSF) and the National Endowment for the Humanities (NEH) to study contemporary language usage in countries where English is mainly and widely used. The dynamic nature of the corpus—with 200 million new words, from 300,000 new articles, added every month—makes it appropriate to study month-by-month change in societal sentiments toward Asians across 8 months, from before the pandemic (October 2019) to during the pandemic (May 2020). Within this study’s timeframe, our corpus consists of 1.5 billion words. This data set is appropriate as Cultivation Theory [18] suggests that the large representation of web-based media within the corpus reflects societal perceptions across 20 countries and provides an extraordinary platform to study the global sentiments of Asians. This unprecedented data set further circumvents the ecological fallacy that limits most survey studies that mistakenly draw country-level conclusions from individual-level surveys. 

#### Data Preprocessing

**Figure 1:**
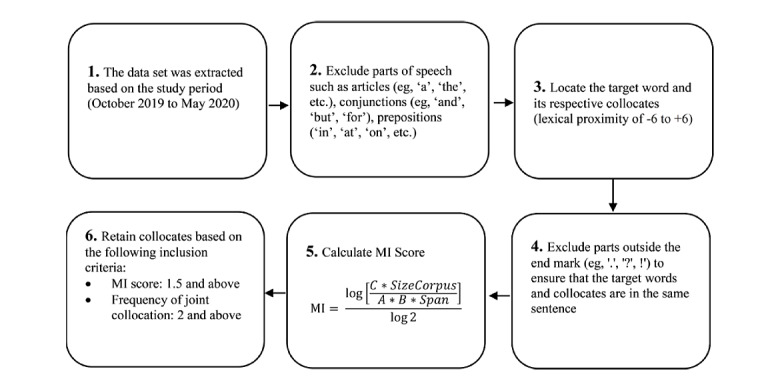
Preprocessing steps to identify collocates of target nouns for sentiment analysis. MI: Mutual Information.

The data set was preprocessed through a 6-step process that aimed at identifying a list of 7 relevant target nouns (*Asian*, *Asians*, *Asia*, *Chinese*, *China*, *Wuhan*, and *Hubei*) and their descriptors (collocates) for sentiment analysis. Figure 1 shows a flowchart for data preprocessing. We generated collocates (ie, words that co-occurred most frequently with each target noun) every month, between October 2019 and May 2020 for each of the 20 countries with the following inclusion criteria: (1) lexical proximity: collocates present within 6 words prior and after the target noun (step 3, Figure 1). Articles such as “the” and “a” were not included in the 6-word lexical span. If the target noun was the first word of a sentence, the collocates from the prior sentence were excluded. (2) Mutual Information (MI) score of 3 and above: collocates had a stronger association with the target noun than with other nouns in the corpus for that country, indicating semantic bonding (step 4, Figure 1) [19]. The MI score estimates word association norms directly from the corpus. It is calculated via sentiment analysis, which shows the MI between collocates and target words. The higher the MI value, the closer the relationship between the collocate and target word. The MI value is calculated using the formula:

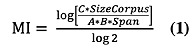



where *A* indicates the possibility of the target word *A* appearing, which is calculated by the frequency of the target word. *B* indicates the possibility of the collocate *B* appearing, which is calculated by the frequency of word *B*. *C* indicates the possibility of *A* and *B* appearing together, which is calculated by the frequency of collocate *B* appearing near the target word *A*. “SizeCorpus” refers to the size of corpus or the number of words. Span is the span of words (eg, if there are 6 words to the left and 6 words to the right of the target word, span=12) [log (2)=0.30103]. This is a well-established application of computational linguistics to study stereotypes in other studies [20-28]. The rigorous process culminated in 85,827 collocates over eight months, across 20 countries.

### Measurement of Societal Sentiments of Asians Across 20 Countries 

After data preprocessing, each collocate was rated on a scale from 1 (very negative) to 5 (very positive) by 2 independent raters with a high interrater reliability (Cronbach α) of .916 (95% CI 0.874-0.958). For instance, *dirty*, *dangerous*, and *suspicious* were rated 1 (very negative); *employee*, *resident*, and *transportation* were rated 3 (neutral); *empowering*, *hero*, and *venerable* were rated 5 (very positive). Table 1 provides sample descriptors associated with narratives on Asians. We calculated a Cumulative Asian Sentiment Score (CASS) per month by taking the respective mean ratings globally to test Hypothesis 1, and by country, to test hypothesis 2. CASS is mathematically defined as follows:

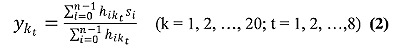



where *Y_kt_* is the CASS for country *K* during month *t*. For country k, during month *t*, *S_i_* is the score of the word (*i*), while *h_ikt_* is the count of the word (*i*).

**Table 1:** Examples of negative, neutral, and positive descriptors of Asians in the media during the COVID-19 pandemic.

Negative	Neutral	Positive
sin	commerce	benefit
weakness	secretary	leading
vile	domestic	calm
protest	affair	rejuvenated
vilify	demand	intelligence
denounce	military	good
deface	philosopher	peaceful
anti-china	deal	intellectual
anger	navy	groundbreaking
punishment	culture	successfully
crisis	market	sophisticated
chaos	country	greatest
agitation	establish	charm
meddling	influence	admiration
discord	rule	improving

### Pandemic Variables

The monthly prevalence of new cases and incidences of new deaths across 20 countries were derived from the Oxford COVID-19 Government Response Tracker [29].

### Hofstede’s Cultural Dimensions

Calculations of the country dimension scores have been reported by Hofstede and Minkov [30], which were based on original cross-national surveys of IBM employees, and subsequent studies (eg, Hofstede Insights, 2020). The country score for each of Hofstede’s dimensions was calculated using this method: First, individual survey responses to each question were calculated at the national level. For questions that were answered on a 5-point Likert scale, the national mean of the responses was calculated. For questions requiring a yes/no or multiple-choice answer, the national percentage of each answer, or a combination of answers (eg, “Option A OR Option C”) was calculated. Next, these national-level scores were combined using a weighted formula to yield a country dimension score based on 3-8 survey questions, resulting in final scores that ranged from 0 to 100.

### Analytical Strategy

Hypothesis 1, positing that societal sentiments toward Asians (as measured with the CASS) have become more negative across 8 months from October 2019 to May 2020, was tested using a trend model to show a significant linear decline in racial sentiments. The linear regression model can be expressed as the following:

*y* = *a* + *bX*

Where *a* represents the intercept, *b* represents the slope and *X* represents the week.

Hypothesis 2, positing that pandemic variables (COVID-19 incidence and mortality rates) and cultural variables (Hofstede’s cultural dimensions) are associated with the CASS, was tested using a linear mixed model. CASS was the independent variable while the pandemic and cultural variables were the dependent variables. The model can be expressed as the following:

*y* = *Xβ* + *Zb* + *ε*

Where *b* ~ *N*(0, *ϕ_θ_*) and *ϵ* ~ *N*(0, *γ_θ_*), X and Z represent model matrices for the fixed effects—pandemic and cultural dimensions—(*β*) and random effects—time—(*b*), respectively. All data preprocessing, text analytics, and statistical analyses were conducted using Python (version 3.7) and OriginPro 2019b.

### Data Accessibility

Data are publicly available at English-Corpora.org.

## Results

### CASS Across 20 Countries During the COVID-19 Pandemic

**Figure 2:**
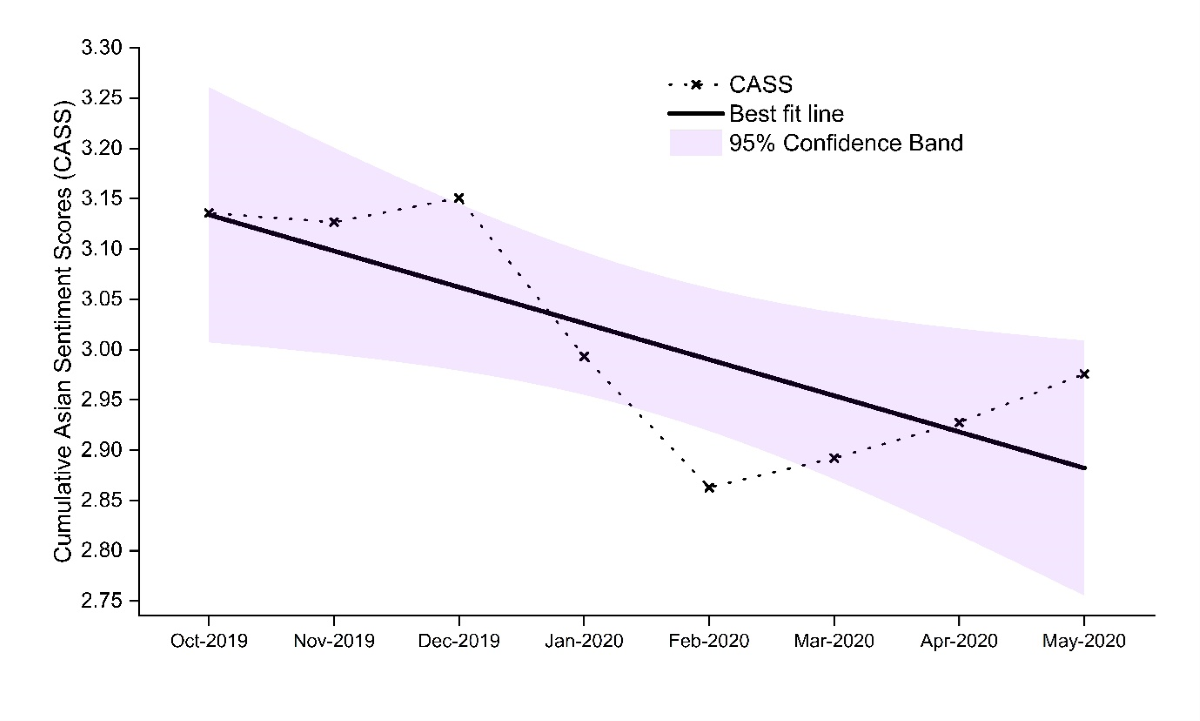
Global Cumulative Asian Sentiment Scores (CASS) across 20 countries from October 2019 to May 2020. Globally, societal sentiments of Asians became more negative across 20 countries—a significant linear decline during the COVID-19 pandemic. CASS were trending neutral before the pandemic (October to November 2019), and plummeted in February 2020. Thereafter, CASS increased gradually, although it has not recovered to prepandemic levels.

**Figure 3:**
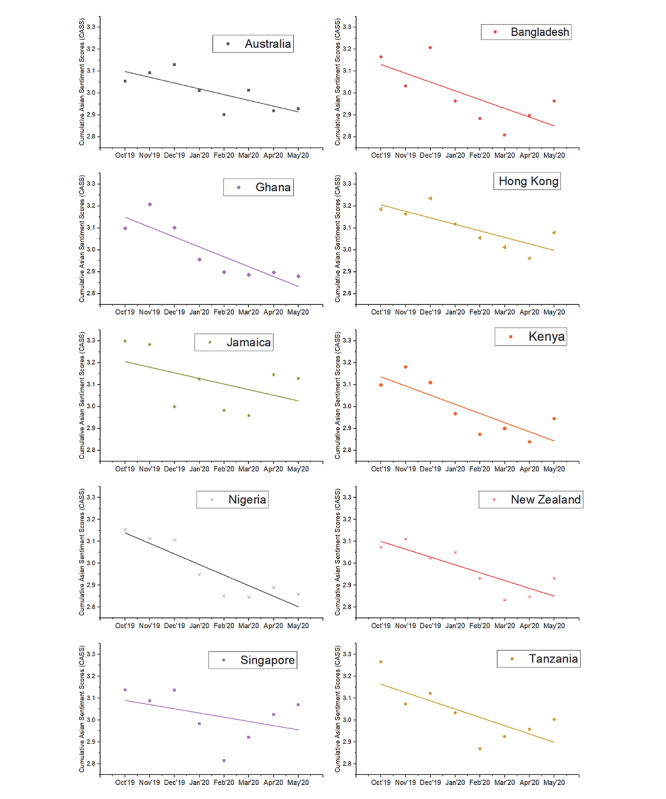
Cumulative Asian Sentiment Scores (CASS) across 10 countries (Australia, Bangladesh, Ghana, Hong Kong, Jamaica, Kenya, Nigeria, New Zealand, Singapore, and Tanzania) from October 2019 to May 2020. There was a significant negative linear trend in CASS across the various countries as the pandemic progressed.

**Figure 4:**
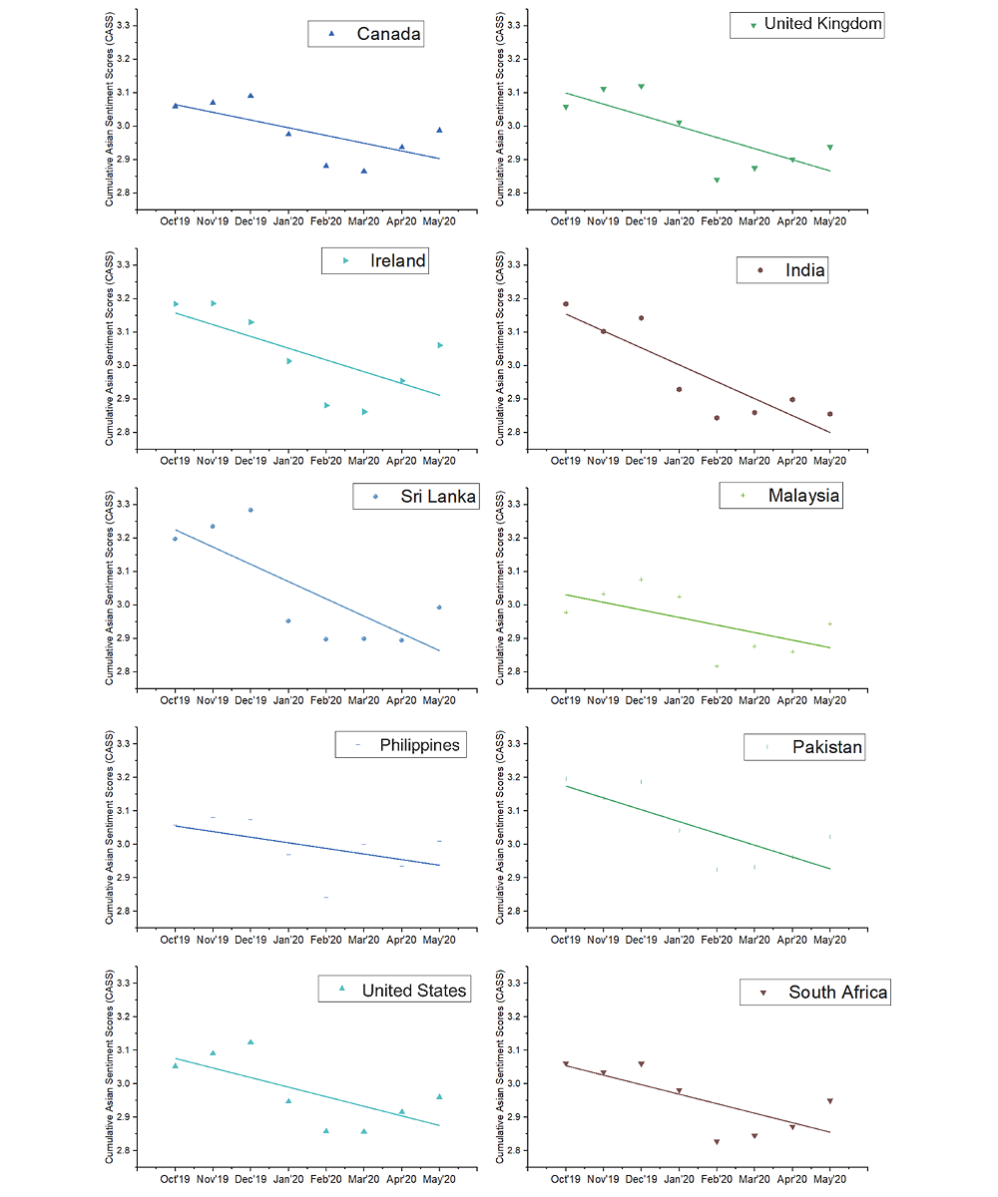
Cumulative Asian Sentiment Scores (CASS) across 10 countries (Canada, the United Kingdom, Ireland, India, Sri Lanka, Malaysia, Philippines, Pakistan, the United States, and South Africa) from October 2019 to May 2020. There was a significant negative linear trend in CASS across the various countries as the pandemic progressed.

Globally, we observed a significant negative linear trend in CASS toward Asians (*β*=–.03, *P*=.018), supporting hypothesis 1 (Figure 2). Other trends such as quadratic and cubic were nonsignificant. Societal sentiments of Asians, measured with the CASS, were trending neutral before the pandemic (October to December 2019) and reached the lowest point in February 2020 during the pandemic. Before the pandemic in December 2019, Jamaica and New Zealand evidenced the most negative societal sentiments toward Asians. In January 2020, when news of the novel coronavirus was released, the United States and Nigeria evidenced the most negative societal sentiments of Asians among 20 countries. The CASS for each of the 20 countries are presented in Figures 3 and 4.

### Predictors of Racial Sentiment Trends During the COVID-19 Pandemic

We tested hypothesis 2 using a mixed model, with pandemic variables (monthly prevalence of new cases and incidence of new deaths for 20 countries) and cultural dimensions as fixed variables, and time as the random variable (Table 2). The pandemic variables of prevalence and mortality rates did not reach significance, while 3 of 4 cultural dimensions were significant: higher power distance (*β=*–.002, *P*<.001), individualism (*β=*–.002, *P*<.001), and uncertainty avoidance (*β*=–.001, *P*<.001) were associated with negativity of societal sentiments toward Asians, holding other covariates constant; this provides support for hypothesis 2.

**Table 2:** Pandemic and cultural predictors of societal sentiments toward Asians across 20 countries during the COVID-19 pandemic (N=28,494): Model 1.^a^ Higher power distance, individualism and uncertainty avoidance were associated with negative sentiments toward Asians; the pandemic variables of prevalence and mortality rates did not reach statistical significance.

Predictor	Value (SE)
Prevalence of new cases^b^	0.000 (0.000)
Incidence of new deaths^c^	0.000 (0.000)
Individualism	–0.002^d^ (0.000)
Power distance	–0.002^d^ (0.000)
Uncertainty avoidance	–0.001^d^ (0.000)
Masculinity	–0.001 (0.000)

^a^*R*^2^=0.737; adjusted *R*^2^=0.723.

^b^Respective monthly COVID-19 incidence rates from October 2019 to May 2020.

^c^Respective monthly COVID-19 mortality rates from October 2019 to May 2020.

^d^*P*<.001.

## Discussion

This is one of the first known empirical studies to dynamically test whether global societal sentiments toward Asians have become more negative month by month from before the pandemic (October 2019) to during the pandemic (May 2020). Our findings show that societal sentiments toward Asians became more negative across the 20 countries over time. This study contributes to the existing literature in 2 ways. First, it contributes to research on race relations by showing how pandemics may have severe repercussions on discourse surrounding certain ethnic groups. Second, by providing insight into the cultural factors associated with anti-Asian sentiments, this study lays the groundwork for more effective cultural communication strategies concerning public health issues.

Historically, incidences of xenophobia have accompanied the onset of global pandemics. With such catastrophic outbreaks, fear often drives those at risk to pin the blame on some group external to their own national, religious, or ethnic identity—in a bid to attribute their troubles to some known entity while reinforcing an “us against them” mentality [31]. As sickness cultivates fear, it can in turn incite moral panic and promote bias. Consequently, minority groups have often found themselves erroneously blamed for being contagious, as others perceive them to be “dirty” [32]. This blame culture could be a driving force for the negativity of societal sentiments toward Asians during the pandemic, as shown in our study.

Delving further, we argue that the negative rhetoric against Asians promoted “otherness” [32-34]. In times of crises such as the current pandemic, it is common for individuals with similar attributes to be categorized as the ingroup, and those who are “different” as the outgroup. The differentiation accentuates the similarities and homogeneity of ingroup members, while juxtaposing the differences of the outgroup members who have been accused of spreading the virus. This reinforces ingroup notions of “normality,” and positions those who are different—the Asians in this context—as deviant, through a process of othering and social exclusion [34]. A consequence of this othering could be prejudice, unfavorable treatment, and discrimination against Asians—possibly leading to heightened racial tensions and frayed social fabric in multiracial communities.

In addition, metaphors are ubiquitous in forming our world view [35]. The adjective “Chinese” in “Chinese virus” is particularly problematic as it metaphorizes the devastation of the pandemic with an ethnicity. Connecting group identities with explicitly negative medical language (ie, virus) serves to categorize those group identities as others. Such “othering” rhetoric only serves to typify stigmatized groups, devaluing them as those in society who possess undesirable characteristics that are outside normal expectations [36].

In relation to hypothesis 2, our results show how anti-Asian sentiments during the COVID-19 pandemic were strongly associated with cultural norms in society and not with the pandemic variables. Three of 4 cultural dimensions [37-42]—individualism, power distance, and uncertainty avoidance—were associated with increasing negativity of racial sentiments. This is one of the first known studies to link cultural dimensions and race relations during the COVID-19 pandemic, and this study attempted to explain these links.

First, we found that increased individualism was associated with negative societal sentiments toward Asians. In societies where tenets of individualism thrive, self-interests and outward expressions of intense emotions such as anger and antagonism [11] are more common, with less importance placed on avoiding confrontation and social harmony. We postulate that high levels of individualism in some societies could have encouraged people to speak their minds and outwardly display of anger and blame toward Asians, resulting in an outpouring of more pernicious sentiments. Moreover, in high-individualism societies, epitomized by a desire for an autonomous identity defined by personal choices and achievements [11], feelings of intrusion and encroachment into these pursuits—inflicted by the virus and those deemed to be “associated” with it—might have also ignited the spark for such confrontational anti-Asian sentiments. We argue that the widespread fear of contracting the disease and thus being precluded from pursuing one’s goals freely engenders prejudice against the outgroup that might be deemed responsible for this, positioning them as effective scapegoats for both the virus and hampering one’s personal individualistic identity.

Second, high power distance was associated with negative societal sentiments toward Asians. In essence, power distance represents the amount of respect and deference between people from different levels of the social hierarchy. Cultures high in power distance advocate a social hierarchy based on institutionalized status differentials, where members from the lower status groups are disadvantaged in terms of resource allocation, are expected to treat their high-status counterparts with deference and respect [11]. We argue that such demarcations of inequality allow for a perpetuation of social dichotomies and an increase in xenophobic sentiments toward those deemed undeserving of respect owing to their perceived “lower” status.

Third, high uncertainty avoidance was associated with negative societal sentiments toward Asians. We suggest that the uncomfortable threat and dangers from stemming those “associated” with the virus, and the pressing need to actively dispel the anxiety of its looming impact, has led to the prevalence of such negative narratives toward Asians. Labeling others on the basis of a variety of stigmas and associating an entire race with the virus not only aids in the identification of “negative” traits that are synonymous with risk, but also reinforces the lack of tolerance for ambiguities and uncertainty associated with particular groups of people who have been deemed to possess deviant behaviors [43]. Such stigmatizing sentiments serve as the cognitive basis of social grouping, helping individuals categorize and cautiously avoid people of particular social groups by displacing the threat of the unknown onto them. We thus argue that uncertainty avoidance is linked to stereotyping, distrusting, and dehumanizing outgroups through harsh sentiments owing to the perceived unpredictability of outgroup members possibly “spreading” the disease and jeopardizing them.

Hofstede’s cultural dimensions have proven to be a useful framework to explore how anti-Asian sentiments have evolved over the course of the COVID-19 pandemic. It is evident that deep-seated cultural norms—individualism, power distance, and uncertainty avoidance—are a driving force in stigmatizing behavior against outgroups and revealed apertures in societal relations that arise during global crises such the COVID-19 pandemic. Future studies should explore the associations between cultural dimensions and racism. In addition, our study limitations could provide ideas for future studies. A key shortcoming is the lack of data from social media in the corpus. The diversity of social media usage across multiple platforms renders data collation challenging, and most social media platforms such as Facebook are closed for public access—they have also become increasingly monetized, selling selected data sets that may not be representative. Though our methodology allows for dynamic social sensing, compared to traditional methods [44-54], this is a significant drawback that we intend to overcome in future studies upon augmenting our database.

The COVID-19 pandemic has deepened fissures in racial relations as posited by numerous commentaries, and now shown empirically by the negativity of societal sentiments of Asians, across 20 countries, over 8 months, from October 2019 to May 2020. It is critical that governments and policy makers consider the cultural underpinnings linked to anti-Asian sentiments when designing appropriate messages for risk communication. In particular, societies high in power distance, individualism and uncertainty avoidance must be prioritized. Confronting the specific cultural factors fueling anti-Asian sentiment will achieve a multiplier effect, eventually forming the basis for solidarity.

## Abbreviations

CASS: Cumulative Asian Sentiment Score
MI: Mutual Information
NEH: National Endowment for the Humanities
NSF: National Science Foundation
